# Cooling and hemodynamic management in heatstroke: practical recommendations

**DOI:** 10.1186/cc5910

**Published:** 2007-05-12

**Authors:** Abderrezak Bouchama, Mohammed Dehbi, Enrique Chaves-Carballo

**Affiliations:** 1 Department of Comparative Medicine MBC-03, King Faisal Specialist Hospital & Research Centre, P.O. Box 3354, Riyadh 11211, Saudi Arabia; 2 Department of Neurosciences MBC-76, King Faisal Specialist Hospital & Research Centre, P.O. Box 3354, Riyadh 11211, Saudi Arabia; 3 Departments of Pediatrics and History and Philosophy of Medicine, Kansas University Medical Center, Kansas, USA

## Abstract

**Introduction:**

Although rapid cooling and management of circulatory failure are crucial to the prevention of irreversible tissue damage and death in heatstroke, the evidence supporting the optimal cooling method and hemodynamic management has yet to be established.

**Methods:**

A systematic review of all clinical studies published in Medline (1966 to 2006), CINAHL (Cumulative Index to Nursing & Allied Health Literature) (1982 to 2006), and Cochrane Database was performed using the OVID interface without language restriction. Search terms included heatstroke, sunstroke, and heat stress disorders.

**Results:**

Fourteen articles reported populations subjected to cooling treatment for classic or exertional heatstroke and included data on cooling time, neurologic morbidity, or mortality. Five additional articles described invasive monitoring with central venous or pulmonary artery catheters. The four clinical trials and 15 observational studies covered a total of 556 patients. A careful analysis of the results obtained indicated that the cooling method based on conduction, namely immersion in iced water, was effective among young people, military personnel, and athletes with exertional heatstroke. There was no evidence to support the superiority of any one cooling technique in classic heatstroke. The effects of non-invasive, evaporative, or conductive-based cooling techniques, singly or combined, appeared to be comparable. No evidence of a specific endpoint temperature for safe cessation of cooling was found. The circulatory alterations in heatstroke were due mostly to a form of distributive shock associated with relative or absolute hypovolemia. Myocardial failure was found to be rare.

**Conclusion:**

A systematic review of the literature failed to identify reliable clinical data on the optimum treatment of heatstroke. Nonetheless, the findings of this study could serve as a framework for preliminary recommendations in cooling and hemodynamic management of heatstroke until more evidence-based data are generated.

## Introduction

Heatstroke is a life-threatening condition characterized by a rapid increase in core temperature to more than 40°C and widespread, multiple organ tissue injury. It is a leading cause of mortality and neurologic morbidity when there is an unaccustomed and sustained increase in climatic temperature [1-4]. During the heat wave that affected Europe in August 2003, there were 14,800 victims in France alone, and 4,277 (28.9%) of these victims were diagnosed as having heatstroke, hyperthermia, or dehydration [4]. As sophisticated climate models predict an increased frequency and severity of heat waves, the incidence of heatstroke with an outcome of mortality or neurologic morbidity is expected to rise if proactive measures are not taken [5,6]. Heatstroke occurs in epidemic form during heat waves, and both hospital emergency department visits and intensive care unit (ICU) admissions increase sharply. Health care professionals should be adequately prepared to promptly recognize and treat this life-threatening illness.

Laboratory studies using cell lines and animal models have established that heat directly induces tissue injury and that the severity of tissue injury and cell death is a function of the degree and duration of hyperthermia [7-10]. Clinical studies have shown that death from heatstroke mostly occurs soon after the onset of hyperthermia and associated cardiovascular failure [11-14]. Up to one third of those victims who survive these initial deleterious effects progress to multi-organ system failure culminating in death or severe neurologic damage [15,16]. The most important objectives in the treatment of heatstroke are, therefore, to decrease body temperature as quickly as possible and to support the cardiovascular system. Achievement of these goals is crucial to the prevention of irreversible organ damage and death.

Effective dissipation of heat is accomplished by increasing the temperature gradient (conduction), water vapor pressure (evaporation), and velocity of air (convection) between the skin and the surrounding air [1,17]. Several techniques have been devised based on these principles, including immersion in cold water, placement of cold packs or ice slush over parts of or the whole body, the use of cooling blankets, and wetting the body surface while continually fanning [16-30]. These cooling techniques have been used for decades, but the evidence supporting their safety and effectiveness in rapidly reaching a safe body temperature and reducing morbidity and mortality has yet to be evaluated.

Acute circulatory failure is found in 20% to 65% of patients with heatstroke and has been implicated in the aggravation of tissue injury and cell death [12,13,16,31,32]. The cause of this failure is not well understood but has been attributed variously to pooling of blood into the cutaneous circulation [31], volume loss by evaporation and insufficient intake of fluid [14,31], myocardial damage [13,33], and distributive shock resembling that of sepsis [34]. Accordingly, several treatment modalities have been proposed without adequate supporting evidence [1,14,34-36]. The objective of this report is to present a systematic review of the literature which addresses these central phases of care, for the purpose of developing evidence-based practice guidelines for cooling and hemodynamic support in heatstroke, especially classic heatstroke.

## Materials and methods

### Search strategy

We searched the National Library of Medicine's Entrez PubMed databases for the period 1966 to April 2006, the CINAHL (Cumulative Index to Nursing & Allied Health Literature) for the period 1982 to April 2006, the Cochrane Database of Systematic Reviews, and the Cochrane Central Register of Controlled Trials Register using the OVID interface. We also manually searched reference lists. The retrieved references were downloaded into a reference manager database, EndNote^® ^version 9 (Thomson, Philadephia, PA/USA). The search was limited to human studies without language restriction and used the MeSH (Medical Subject Heading) terms heatstroke, sunstroke, and heat stress disorders.

### Selection criteria

Two of the authors independently evaluated the retrieved articles and made selections based on the population, intervention, outcome, and study design.

#### Cooling methods

We examined adult and pediatric populations who had classic or exertional heatstroke and who were subjected to cooling treatment in studies that reported cooling time and neurologic morbidity or mortality as endpoints. To be eligible for review, the study had to report original data and consist of randomized controlled studies or observational studies (cohort or descriptive studies, case-control, and case series) involving more than 10 patients.

Exclusion criteria included (a) studies reporting only biochemical and/or immunological endpoints (that is, clinical chemistry, hormones, cytokine levels, and immune cell responses), (b) heat stress disorders (that is, occupational or induced whole-body hyperthermia), (c) reviews, case reports, and case series of fewer than 10 patients, and (d) experimental studies using healthy volunteers or animal models.

#### Hemodynamic management

Adult and pediatric populations with classic or exertional heatstroke who were monitored invasively with central venous or pulmonary artery catheters and reporting right- or left-filling pressures or cardiac output as endpoints were examined. To be eligible, the studies must have reported original data in more than five patients.

#### Endpoints and definitions

Heatstroke is defined as a core body temperature rising to more than 40°C and central nervous system abnormalities such as delirium, convulsions, and/or coma resulting from exposure to a high environmental temperature (classic or non-exertional heatstroke) or strenuous physical exercise (exertional heatstroke). Table 1 presents common and distinctive features of classic and exertional heatstroke [1]. Cooling is defined as physical methods or pharmacologic agents aimed at accelerating cooling to a predefined target temperature. Neurologic morbidity is defined as sustained central nervous system abnormalities such as delirium, convulsions, and coma following cooling and/or during long-term follow-up in survivors.

**Table 1:** Common and distinctive features of classic and exertional heatstroke

Features	Classic	Exertional
Common		
Hyperthermia	> 40°C	> 40°C
Central nervous system alteration	Delirium, convulsion	Delirium, convulsion
Hypotension	20%–30%	Unknown
Distinctive		
Age	Elderly	Young
Skin	Hot, dry	Hot, profuse sweating
Rhabdomyolysis	Mild/moderate	Severe
Renal failure	Uncommon	Common
Lactic acidosis	Mild/moderate	Severe
Glycemia	Hyperglycemia	Hypoglycemia
Disseminated intravascular coagulation	Mild/moderate	Severe

## Results

### Search results

The search identified 926 papers on heat illnesses. From these, four randomized controlled studies [26,28-30] and 10 observational studies met the eligibility criteria for the evaluation of cooling methods [16,18-25,27]. Seven studies that used cooling method based on conduction were identified, five on evaporation and two on pharmacologic cooling. Various target temperatures ranging from 37°C to 40.1°C for safe discontinuation of cooling were used. Five observational studies met the criteria for the assessment of hemodynamic management [31,34,37-39]. The total number of patients reported in these 19 publications was 556, and these were subjected to further analysis (Tables 2, 3, 4, 5, 6).

**Table 2:** Summary of data on cooling methods based on conduction in the treatment of exertional heatstroke

Study (country, year)	Population	Study design	Intervention	Outcomes measured	Results	Limitations
[18] (Israel, 1967)	Exertional heatstroke (*n *= 36)	Case series	Ice-filled rubber bottles over the whole body; cool air-conditioned room; target T_rect_: not given	Mortality; morbidity	Mortality: 22.2%; neurologic morbidity: 11.1%	Patients enrolled over 10-year period; no cooling time provided; cooling performed in different centers
[19] (U.S., 1975)	Exertional heatstroke (*n *= 15)	Case series	Iced water immersion; target T_rect_: 38.8°C	Mortality; morbidity	Mortality: 0%; neurologic morbidity: 0%	None
[20] (U.S., 1975)	Exertional heatstroke (*n *= 13)	Case series	Iced water immersion; target T_rect_: 38.3°C	Cooling time; mortality; morbidity	Cooling time: < 60 minutes, 92.3%; cooling time: > 60 minutes, 7.7%; mortality: 0%; neurologic morbidity: 0%	None
[21] (U.S., 1979)	Exertional heatstroke (*n *= 13)	Case series	Iced water immersion; target T_rect_: 38.3°C to 38.8°C	Cooling time; mortality; morbidity	Cooling time (range): 10 to 40 minutes; myocardial ischemia: 7.7%; neurologic morbidity: 0%; mortality: 0%	None
[30] (U.S., 1996)	Exertional heatstroke (*n *= 21)	Randomized controlled trial	Iced water immersion (1°C to 3°C) torso and upper legs (*n *= 14) versus wet towel and air exposure at 24.4°C (*n *= 7); target T_rect_: 38.2°C to 40.1°C	Cooling rate	Conductive-based cooling faster than evaporative (0.20 ± 0.02 versus 0.11 ± 0.02°C/minute)	Small sample size; comparability of baseline characteristics undetermined; randomization method not specified; evaporative technique suboptimal

T_rect_: rectal temperature.

**Table 3:** Summary of data on cooling methods based on conduction in the treatment of classic heatstroke

Study (country, year)	Population	Study design	Intervention	Outcomes measured	Results	Limitations
[16] (U.S., 1982)	Classic heatstroke (*n *= 28)	Case series	Iced water immersion; brisk massage with ice; target T_rect_: ≤38.9°C	Cooling time; mortality; morbidity	Cooling time: < 30 minutes, 93%; cooling time: 30 to 45 minutes, 7%; mortality: 14.3%; neurologic morbidity: 14.3%	Patients switched to brisk massage were not identified
[24] (U.S., 1986)	Classic heatstroke (*n *= 39)	Case series	Ice packs to axilla and groin; cold wet sheets applied to torso; ice water lavage; cooling blankets; target T_rect_: ≤38.9°C	Cooling time; mortality	Cooling time: < 60 minutes, 69%; mortality: 15%; cooling time: > 60 minutes, 31%; mortality: 33%;	Retrospective assignment of group; comparability of the groups at baseline questionable

T_rect_: rectal temperature.

**Table 4:** Summary of data on cooling methods based on evaporation in the treatment of classic heatstroke

Study (country, year)	Population	Study design	Intervention	Outcomes measured	Results	Limitations
[25] (U.S., 1986)	Classic heatstroke (*n *= 14)	Case series	Ice to the lateral aspect of the trunk and spraying of tepid water (40°C); fan directed to patients; massage to torso and neck; chilled intravenous solution; target T_rect_: ≤ 39.4°C	Cooling time; mortality; morbidity	Median (range) cooling time: 60 minutes (34 to 89 minutes); mortality: 7.1%; neurologic morbidity: 0%	Combination of several cooling techniques; relative contribution of each difficult to ascertain
[27] (Saudi Arabia, 1987)	Classic heatstroke (*n *= 25)	Case series	Wet gauze sheet with water at 20°C; fan with speed airflow of 2.6 m/s; target T_rect_: ≤ 39°C	Cooling time; mortality; morbidity	Mean (range) cooling time: 40.4 minutes (20 to 145 minutes); mortality: 0%; morbidity: 24%	No follow-up
[22] (Kuwait, 1980)	Classic heatstroke (*n *= 18)	Case series	Body cooling unit*; target T_rect_: < 38°C	Cooling time; mortality;	Cooling time: 26 to 300 minutes; mortality: 11.1%	No follow-up
[23] (Kuwait, 1981)	Classic heatstroke (*n *= 174)	Case series	Body cooling unit*; target T_rect_: < 38°C	Cooling time; mortality;	Mean (range) cooling time: 78 minutes (20 to 180 minutes); mortality: 14.9%	No follow-up
[26] (Saudi Arabia, 1986)	Classic heatstroke (*n *= 16)	Randomized controlled trial	Evaporative cooling using body cooling unit* (*n *= 8) versus conventional method (wet gauze sheet with water at 25°C and fanning air at 20°C) (*n *= 8); body cooling unit*; target T_rect_: ≤ 38.5°C	Cooling time; mortality; morbidity	No significant difference in cooling time; no death in either group; neurologic morbidity: 25% versus 12.5%	Small sample size; randomization method not specified; no follow-up

T_rect_: rectal temperature.

*A special bed preset to spray atomized water at 15°C and warm air at 45°C over the whole body surface to keep the wet skin temperature between 32°C and 33°C [40].

**Table 5:** Summary of data on pharmacologic cooling in the treatment of classic heatstroke

Study (country, year)	Population	Study design	Intervention	Outcomes measured	Results	Limitations
[28] (Saudi Arabia, 1990)	Classic heatstroke (*n *= 20)	Randomized controlled study	Evaporative cooling + dantrolene 2 to 4 mg/kg IV (*n *= 8) versus evaporative cooling alone (*n *= 12); target T_rect_: ≤ 38.9°C	Cooling time; mortality; morbidity	Cooling time in the dantrolene group lower than control (49.7 ± 4.4 versus 69.2 ± 4.8 minutes; *p *< 0.01); no difference in morbidity and mortality	Small sample size; randomization method not specified; comparability of baseline characteristics questionable
[29] (Saudi Arabia, 1991)	Classic heatstroke (*n *= 52)	Randomized controlled study	Evaporative cooling + dantrolene 2 mg/kg IV (*n *= 26) versus evaporative cooling + placebo (*n *= 26); target T_rect_: ≤ 39.4°C	Cooling time; organ dysfunction; length of hospital stay; mortality	No significant difference between study and control groups for any of the endpoints	None

IV, intravenous; T_rect_: rectal temperature.

**Table 6:** Summary of data on hemodynamic monitoring and support in heatstroke

Study (country, year)	Population	Intervention	Outcomes measured	Results
[34] (U.S., 1972)	Exertional heatstroke (*n *= 8)	Pulmonary artery catheter; fluid therapy	Hemodynamic profile; response to fluid therapy; mortality	Hyperdynamic profile, *n *= 7; hypodynamic profile, *n *= 1; optimal response to fluid: 1,200 ml per 4 hours and cooling; mortality: 0%
[31] (U.S., 1979)	Classic heatstroke (*n *= 7)	Pulmonary artery catheter; fluid therapy	Hemodynamic profile; response to fluid therapy; mortality	Hyperdynamic profile, *n *= 2; hypodynamic profile, *n *= 5; failure to respond to fluid: 6,000 ml per 24 hours and cooling; no pulmonary edema; mortality: 71%
[37] (Saudi Arabia, 1989)	Classic heatstroke (*n *= 13)	Pulmonary artery catheter; fluid therapy	Hemodynamic profile; response to fluid therapy; mortality	Hyperdynamic profile, *n *= 13; fluid 400 to 1,200 ml per 4 hours, *n *= 8, no pulmonary edema; fluid 1,200 to 1,800 ml per 4 hours, *n *= 5, pulmonary edema; mortality: 7.6%
[38] (Saudi Arabia, 1993)	Classic heatstroke (*n *= 10)	Pulmonary artery catheter	Hemodynamic profile; mortality	Hyperdynamic profile, *n *= 8; hypodynamic profile with normal systemic vascular resistance, *n *= 1; normodynamic profile, *n *= 1; mortality: 10%
[39] (Saudi Arabia, 1991)	Classic heatstroke (*n *= 34)	CVP monitoring; fluid therapy	CVP; response to fluid therapy; mortality	CVP < 3 cm H_2_O, *n *= 12 (35.3%); CVP 3 to 10 cm H_2_O, *n *= 16 (47%); CVP >10 cm H_2_O, *n *= 6 (17.6%); fluid 500 to 2,500 ml titrated to CVP (3 to 8 cm H_2_O); optimal response, no pulmonary edema; mortality: 0%

CVP, central venous pressure.

### Cooling methods based on conduction

Conduction is the passive transfer of heat from the body into the surroundings air, liquid, or solid in contact with the skin along a temperature gradient.

#### 1. Exertional heatstroke

##### Immersion in iced water

This is the most used conventional cooling technique and involves placing the patient in a tub of iced water and continuously massaging the extremities to promote vasodilatation and heat loss [11,12,16,19,21]. Four studies that used this method in patients with exertional heatstroke were identified [19-21,30]. Table 2 presents a summary of data on cooling methods based on conduction in the treatment of exertional heatstroke.

Three of the studies included 41 young military personnel treated with immersion in iced water to a target temperature of between 38.3°C and 38.8°C [19-21] (Table 2). The cooling time ranged from 10 to 60 minutes in all patients but one. No fatalities were reported. Neurologic morbidity, characterized by marked confusion, violent behavior, and frank psychosis, was present during recovery but subsided subsequently [20].

The fourth study was a prospective comparison of immersion of the torso and thighs in iced water (1°C to 3°C), with evaporative cooling using wet towels and exposure to air at 24.4°C without fan ventilation, in hyperthermic long-distance runners [30]. The immersion technique cooled twice as fast as the evaporative technique. Morbidity, mortality, and follow-up were not reported. The assignment of patients to each arm of treatment was not randomized, and the evaporative cooling technique was not optimal (Table 2).

##### Application of cold packs

One study in which 36 patients were treated with cold packs applied to the whole body was identified. No cooling time was provided, but mortality and neurologic morbidity in survivors were 22.2% and 11.1%, respectively [18] (Table 2).

#### 2. Classic heatstroke

Table 3 presents a summary of data on cooling methods based on conduction in the treatment of classic heatstroke.

##### Immersion in iced water

This was applied to 28 patients of a mean age of 71 years (range, 47 to 90 years) with associated comorbid illnesses [16]. The cooling rate achieved was comparable with that of the younger and healthier population described above; however, 14.3% of the patients died and another 14.3% sustained severe brain damage. The technique was poorly tolerated and had to be converted to ice massage in some patients, who were not further identified.

##### Other cooling methods based on conduction

These included non-invasive and invasive techniques. The former comprised the use of cooling blankets or ice or cold packs covering all or parts of the body, commonly in proximity to large vessels (that is, neck, groin, and axillae) [1]. The invasive techniques consisted of administration of chilled intravenous solution and iced gastric, colonic, bladder, or peritoneal lavage.

A single study that consisted of 39 patients treated with cold packs was identified [24] (Table 2). Thirty-one of the 39 patients had cold packs applied to the axillae and groin and cold wet sheets applied to the trunk; this was combined with cooling blankets in four patients and with ice water lavage in five patients. The overall mortality rate was 20.5%. A cooling time of less than 60 minutes was achieved in 27 patients (69%) with a mortality rate of 15%, whereas in the group with a longer cooling time, the mortality rate was 33%. Although the difference was not statistically significant, this observation suggests that rapid cooling may be an important determinant of outcome. There were insufficient data to assess the value of invasive cooling techniques.

### Cooling methods based on evaporation

Evaporative cooling is based on the physical principle that the conversion of 1.7 ml of water to a gaseous phase consumes 1 kcal of heat [1,11]. The efficiency of evaporative cooling depends on a high water-vapor pressure gradient accomplished by continuously spraying the skin with water and blowing with hot air to keep it warm [1,17].

#### Exertional heatstroke

Other than the study mentioned above [30], no study describing the use of the evaporative cooling technique in exertional heatstroke was found (Table 2).

#### Classic heatstroke

Five studies comprising 247 patients treated by evaporative cooling techniques, either conventional or by using a specially designed cooling bed, were identified [22,23,25-27]. Table 4 presents a summary of data on cooling methods based on evaporation in the treatment of classic heatstroke.

##### Conventional evaporative cooling

This consists of applying gauze sheets wetted with water at 20°C to 40°C and fanning air at room temperature. In a case series of 14 patients of a mean age of 66 years and who had associated comorbid illnesses, cooling by evaporation using water at 40°C and fan ventilation enabled cooling in 34 to 89 minutes, with only one fatality and no morbidity in survivors [25]. In this study, the evaporative method was combined with conductive techniques, namely cooling blanket, gastric, colonic and bladder lavage with iced saline, and intravenous administration of chilled solutions; thus the relative contribution of each modality was difficult to assess.

In another series (*n *= 25 patients) using a similar method but applying wet gauze at 20°C, the cooling time ranged from 20 to 145 minutes, with no mortality. Six (25%) patients progressed to dysfunction of one or more organs with no further follow-up [27].

##### Evaporative cooling using body cooling unit

The body cooling unit (BCU) is a bed specially constructed to combine spraying of atomized water at 15°C and blowing of hot air at 45°C over the whole body surface to keep the wet skin temperature between 32°C and 33°C [40]. The BCU has been used extensively during the Muslim pilgrimage to Makkah, Saudi Arabia, in the summer months, when the incidence of heatstroke rises markedly [23]. A total of three studies using the BCU were identified [22,23,26] (Table 4).

The first two studies comprised 192 patients suffering from classic heatstroke [22,23]. The cooling time to reach a target temperature of 38°C ranged from 26 to 300 minutes (mean, 78 minutes). The mortality rate varied between 11.1% and 14.9%. No neurologic morbidity post-cooling was observed among survivors.

The third was a controlled study that compared conventional evaporative cooling with cooling using the BCU [26]. The small sample size precluded any meaningful interpretation of the data.

### Cooling methods based on medications

Dantrolene sodium is a skeletal muscle relaxant that reduces muscular heat produced during abnormally sustained contraction such as observed in malignant hyperthermia and neuroleptic malignant syndrome [28,29,41]. It acts directly on the skeletal muscle and is thought to inhibit calcium release from the sarcoplasmic reticulum to the cytosol during sustained contraction and thereby reverses muscle rigidity and decreases production of heat [41,42]. Table 5 presents a summary of data on pharmacologic cooling in the treatment of classic heatstroke. Two randomized controlled studies assessed the cooling enhanced pharmacologically by using dantrolene sodium [28,29].

In a randomized study of 20 patients, 2 to 4 mg/kg dantrolene sodium plus evaporative cooling was found to reduce significantly the cooling time compared with evaporative cooling alone [28]. However, flaws in the study design (namely, a small number of patients and an undefined randomization procedure with the use of different cooling techniques and doses of dantrolene sodium, which were non-blinded to clinicians) raise doubts about the scientific validity of the results.

In contrast, the second study of 52 patients was double-blinded, randomized, and adequately powered to demonstrate a 30-minute difference in cooling time. This study showed that 2 mg/kg dantrolene sodium was ineffective in reducing the cooling time, length of hospital stay, and mortality (Table 5) [29].

Antipyretic drugs were used following the findings of increased pyrogenic cytokines during heat stress [1]. These were given to few patients with heatstroke and concomitantly with other cooling techniques, and thus their effectiveness could not be properly assessed [15].

### Hemodynamic support

The hemodynamic response to heat stress has been well studied both in supine, resting, healthy volunteers heated to the limits of thermal tolerance and during exercise in a hot environment [43]. The circulatory adjustments were comparable but differed in magnitude and muscular perfusion, which were more marked for the latter. These changes included a marked increase in cardiac output accompanied by redistribution of blood flow to the cutaneous circulation (up to 50% of cardiac output) at the expense of renal and splanchnic circulation, while total peripheral vascular resistance remained unchanged [43]. Studies in animal experiments suggest that secondary splanchnic vasodilation mediated by local production of nitric oxide results in cardiovascular collapse and hyperthermia [44,45]. In contrast, the hemodynamic alterations that follow heatstroke have not been completely elucidated [31,34,37-39].

The search strategy used for this review yielded five studies on the hemodynamic alterations in heatstroke with monitored response to therapy [31,34,37-39]. Table 6 presents a summary of data on hemodynamic monitoring and support in heatstroke.

#### Hemodynamic alterations in exertional heatstroke

O'Donnell and Clowes [34] performed serial hemodynamic measurements in eight marine soldiers suffering from acute exertional heatstroke. Seven of the patients displayed an elevated cardiac index and low systemic vascular resistance. In one patient, cardiac index was low and systemic and pulmonary vascular resistances were elevated with a marked increase in right atrial pressure (Table 6).

#### Hemodynamic alterations in classic heatstroke

By means of right heart catheterization, the hemodynamic profile of 30 elderly patients suffering from classic heatstroke was investigated in three studies [31,37,38] (Table 5). Twenty-three (76.6%) of the patients exhibited a hyperdynamic profile, and 6 (20%) a hypodynamic profile. The clinical response to fluid therapy and the risk of pulmonary edema varied among studies, thus precluding any meaningful interpretation.

In the last study, the state of hydration and response to a conservative fluid challenge were prospectively assessed with central venous pressure (CVP) monitoring in 34 consecutive patients with classic heatstroke [39]. Twelve patients had a CVP reading of zero or less on arrival, and eight of these patients presented in shock state. Administration of an average of 1 liter (0.5 to 2.5 liters) of crystalloids titrated to a CVP of 3 to 8 cm H_2_O restored an optimal hydration state and did not result in any signs of fluid overload.

## Discussion

### Cooling methods

The present study evaluated various cooling techniques used to treat heatstroke. We made the following observations:

First, consistent with a previous systematic review, the cooling method based on conduction, namely immersion in iced water started within minutes of the onset of exertional heatstroke, was fast, safe, and effective in young, healthy, and well-trained military personnel or athletes [19-21,30,46]. Furthermore, when extending the analysis to classic heatstroke, this study demonstrated that immersion in iced water of elderly patients suffering from classic heatstroke had a comparable efficacy in achieving a high cooling rate, but the technique was poorly tolerated and was associated with increased morbidity and mortality [16]. These findings concurred with those of earlier studies in which severe shivering, agitation, and combativeness required the mobilization of a large number of staff for restraint and in which sedation was necessary [11,12,20]. Other drawbacks reported were poor hygiene (heatstroke is often associated with vomiting and diarrhea) and difficulty both in achieving optimal monitoring and resuscitating unconscious and hemodynamically unstable patients [11,20].

Second, although none of the randomized controlled studies compared evaporative with conductive cooling methods in patients with classic heatstroke, the cooling methods based on evaporation appeared to be less efficient than immersion in iced water in dissipating heat, but they were well tolerated [22,23,25,26,28,29]. Despite a slower cooling rate, the mortality rate was low, ranging from 0% to 14.9% [22,23,25,26,28,29]. For many reasons – such as heterogeneity of the population studied, lack of information on the time required to recognize heatstroke and initiate cooling, and comparability of supportive management – how this favorable outcome compared with that of cooling by immersion in iced water is difficult to ascertain. Until randomized controlled studies comparing these two modalities of cooling treatment are performed, each should be considered an equivalent option in the treatment of classic heatstroke. Perhaps the final choice should depend on the patient's condition, the availability of equipment, and the staff's familiarity with the selected technique.

Third, our review showed that non-invasive and well-tolerated cooling modalities, such as ice packs or cold packs, wet gauze sheets, and fan alone or in combination, could represent reasonable alternatives since these are easily applied and readily accessible during epidemic classic heatstroke, when a large number of frail elderly patients are seen in the emergency room [24-27]. Indeed, in four studies, the cooling time using these techniques in patients with classic heatstroke was reasonably low and the outcome was acceptable [24-27].

Fourth, this review suggested that pharmacologic treatment (namely, dantrolene sodium as an adjunct to physical methods to accelerate cooling) was ineffective, whereas antipyretic agents were not properly assessed [28,29]. Antipyretics such as aspirin and acetaminophen should be avoided because of their potential to aggravate the coagulopathy and liver injury of heatstroke.

Fifth, our review found no evidence for a specific endpoint temperature at which to halt cooling. A rectal temperature of 39°C or less appeared to be safe in terms of mortality in most of the studies, but associated long-term morbidity (particularly neurologic) has not yet been established and further study is required.

### Hemodynamic management

Although rapid and effective cooling is the cornerstone of treatment, the management of circulatory failure in heatstroke is also important [12-14,16]. In an earlier study of 100 patients with classic heatstroke, Austin and Berry [12] showed that hypotension was associated with a mortality rate of 33% compared with 10% in patients without hypotension. Hart and colleagues [16] found that the necessity for supplementary vasoactive treatment to restore blood pressure was associated with both a high mortality rate and neurologic disability. These observations were reinforced by a recent survey of 345 patients with classic heatstroke which demonstrated that the use of vasoactive drugs within the first 24 hours of admission to the ICU was independently associated with an increased risk of death [32]. These findings established a link between hypotension and poor outcome, suggesting that prevention and treatment of the hemodynamic instability of heatstroke may contribute to improved outcome.

Based on available data, the present study established the following evidence:

The circulatory alterations and collapse in both exertional and non-exertional heatstroke were, for the most part, due to a form of distributive shock characterized by vasodilatation and relative or absolute hypovolemia [31,34,37,38]. A hypodynamic state was observed in approximately 20% of the patients [31,38]. Although myocardial failure appeared only rarely, the presence of myocardial dysfunction at the onset of heatstroke seemed more difficult to ascertain in an elderly population with a high prevalence of pre-existing coronary or structural cardiac diseases [33,34,47,48]. Overall, the findings of our study suggested that the hemodynamic profile of heatstroke shares many similarities with sepsis and is consistent with the systemic inflammatory response demonstrated in human and experimental heatstroke [1,49].

In contrast to the findings on the hemodynamic profile, the data on the risk of pulmonary edema were inconclusive. The varying amount of fluid administered in different studies did not explain why some patients developed pulmonary edema and others did not. There were numerous confounding factors such comorbid illness, acute lung injury, and/or heat-related myocardial damage that may be associated with heatstroke and could have accounted for this difference.

Although the present systematic review showed that hypotension could impact negatively on outcome, there was even less evidence to support the concept that restoration of blood pressure would ameliorate the outcome. The findings of this review suggested that besides cooling, the initial hemodynamic management in both exertional and classic heatstroke should include fluid replacement sufficient to restore blood pressure and tissue perfusion. Supporting evidence, however, is lacking for more specific recommendations, such as the selection of a specific type of fluid and the rate and volume of infusion, and so careful fluid replacement is recommended as the incidence of pulmonary edema during resuscitation of heatstroke appeared to be high in some studies [37-39]. Until new evidence is established, the therapeutic approach recommended for hemodynamic management of sepsis can also be applied to heatstroke because of the pathophysiological similarities between the two diseases [50]. Fluid resuscitation should be titrated to clinical endpoints of optimal heart rate, urine output, and blood pressure, and the patients who remain hypotensive after initial fluid and cooling therapy should be considered for invasive hemodynamic monitoring.

### Limitations

This review identified the lack of reliable data from well-designed controlled studies that address this important phase of emergency treatment of heatstroke, namely cooling and hemodynamic management. Therefore, the findings and recommendations suggested above should be taken cautiously because they were derived mostly from observational case-series studies without control groups and involved a heterogeneous population, with the probable presence of other confounding factors.

### Future directions

This study showed that most of the cooling techniques used in the treatment of heatstroke were outdated and rudimentary, whereas a new generation of cooling devices is now available following the findings that induced hypothermia may be beneficial in patients with neurologic injury, particularly post-cardiac arrest [51]. These innovative cooling techniques and devices, which comprise infusion of large volumes of ice-cold crystalloid fluid (4°C), cooling catheters using ice-cold fluids circulating in a closed circuit, cooling helmets designed to cool the brain, and cold-air or water pads and blankets controlled with sophisticated algorithms, should prove to be of some benefit to patients with heatstroke [51]. However, their efficacy must be rigorously tested in hyperthermic patients and not simply extrapolated from studies on induced hypothermia. Humans regulate heat exchange with the environment by modulating the blood flow through the cutaneous circulation. Indeed, hyperthermia is a high blood flow state due to hypothalamus-mediated cutaneous vasodilatation, which is very different from the familiar low blood flow profile observed in post-cardiac arrest [43,51].

In the past decade, there has been substantial advance in the understanding of the mechanisms of heatstroke injury at the molecular and cellular levels [1]. In addition to direct cytotoxicity, it is suggested that heat triggers a complex pathophysiology that involves alteration of heat shock responses, exaggeration of the acute-phase response, and excessive activation of coagulation [1]. Normalizing the body temperature with cooling may not be enough to abrogate the inflammation, coagulation activation, and progression to multiple organ dysfunction and death in more than a third of patients [1,15,52,53]. Therefore, in addition to improving the cooling techniques, it is necessary to develop therapy based on modulation of the inflammatory and coagulation responses [54-57]. Immunomodulators such as interleukin-1 receptor antagonists, corticosteroids, and recombinant activated protein C improve survival in the animal model of heatstroke but have yet to be studied in humans [54-57].

## Conclusion

This review revealed the need for more conclusive research aimed at identifying the optimal cooling methods and hemodynamic management of heatstroke. Although the recommendations suggested should be taken cautiously, they were based on a thorough review of the available evidence and hence reflect the current state of knowledge. Until further evidence is established, these could serve as a practical approach for the cooling and hemodynamic management of heatstroke, a condition predicted to become more frequent in epidemic form in the near future.

## Key messages

• Rapid cooling and management of circulatory failure are crucial to the prevention of irreversible tissue damage and death in heatstroke.

• The literature review failed to identify reliable clinical data on optimum cooling and hemodynamic management of heatstroke.

• Immersion in iced water is effective among young people, military personnel, and athletes with exertional heatstroke.

• No evidence to support the superiority of any one cooling technique in classic heatstroke was found.

• The circulatory alterations in heatstroke were mostly due to a form of distributive shock associated with relative or absolute hypovolemia.

## Abbreviations

BCU = body cooling unit; CVP = central venous pressure; ICU = intensive care unit.

## Competing interests

The authors declare that they have no competing interests.

## Authors' contributions

AB made substantial contributions in the conception, design, acquisition, analysis, and interpretation of data. MD participated in the acquisition and analysis of data. EC-C participated in the conception and design of data. All authors drafted and revised the manuscript and have given final approval of the version to be published.
